# Cardiac manifestations and effects of enzyme replacement therapy for over 10 years in adults with the attenuated form of mucopolysaccharidosis type I

**DOI:** 10.1016/j.ymgmr.2020.100662

**Published:** 2020-10-20

**Authors:** Kenta Sugiura, Toru Kubo, Yuri Ochi, Yuichi Baba, Takayoshi Hirota, Naohito Yamasaki, Hiroaki Kitaoka

**Affiliations:** Department of Cardiology and Geriatrics, Kochi Medical School, Kochi University, Kochi, Japan

**Keywords:** Mucopolysaccharidosis, Scheie syndrome, Enzyme replacement therapy, Adults, Valvular disease

## Abstract

**Background:**

Mucopolysaccharidosis type I (MPS I) is a rare autosomal recessive disease caused by a deficiency of the lysosomal enzyme α-L-iduronidase. Cardiac manifestations such as valvular heart disease are associated with poor prognosis. There have been only a few reports on the effect of long-term enzyme replacement therapy (ERT) for adult patients with the attenuated form of MPS I (Scheie syndrome) and cardiac involvement.

**Methods:**

We retrospectively reviewed four adult patients of Scheie syndrome for which ERT was performed in our hospital. We investigated the findings of electrocardiography and echocardiography for the four patients performed before and 10 years after the initiation of ERT to evaluate the efficacy for ERT in Scheie syndrome.

**Results:**

The ages of the patients at the initiation of ERT ranged from 26 to 46 years. The mean follow-up period was 129 months (121 to 134 months). Two patients underwent valve replacement surgery before the initiation of ERT. One patient had gradual progressive aortic valve stenosis and mitral valve stenosis during the course of ERT, and double valve replacement was finally performed. The patient who had started ERT at the youngest age did not develop significant cardiovascular disease. Regarding clinical courses with ERT for a period of 10 years, all four patients survived and they showed relatively stable cardiac conditions although two patients developed sick sinus syndrome after the valvular surgery.

**Conclusions:**

Valvular disease in patients with Scheie syndrome occur at a young age. In a limited number of the four patients, ERT might contribute the stability of cardiac condition.

## 1 Background

Mucopolysaccharidosis type I (MPS I) is a lysosomal storage disorder with an autosomal recessive inheritance pattern caused by a deficiency of the enzyme α-L-iduronidase (α-IDUA) that results in the accumulation of glycosaminoglycans (GAGs). MPS I has traditionally been classified into three syndromes (Hurler syndrome, Hurler-Scheie syndrome, and Scheie syndrome). Hurler syndrome is a most severe form of MPS I and most of the patients with this form of MPS I die by the age of 10 years. Although patients with Hurler-Scheie syndrome have the same medical problems as those in patients with Hurler syndrome, the rate of progression is slower. Patients with Hurler-Scheie syndrome have little or no cognitive impairment and die in their teens or twenties. Patients with Scheie syndrome, an attenuated type of MPS I, have less extensive disease and a potentially normal life span. Accumulation of GAGs affects multiple organ systems and leads to multiple manifestations. The involvement of cardiovascular system such as valvular heart disease, left ventricular (LV) hypertrophy, and pulmonary hypertension is common in MPS I patients [1,2]. Particularly, cardiac valve abnormalities were often observed in 67.7% of Scheie patients [3]. Consequently, cardiac disease can be a cause of premature death in patients with Scheie syndrome. However, there has been no natural cohort available for cardiac outcome in those patients.

Enzyme replacement therapy (ERT) has been approved for MPS since the 21th century [4]. Human α-IDUA therapy reduces the levels of lysosomal storage and results in some clinical improvements. Theoretically, it might be difficult to prevent the progression of pre-existing cardiac disease because of irreversible changes in the delicate structures of heart valves and chordae tendineaes inflicted by years of GAG accumulation [5]. On the other hand, Laraway et al. reported that valvular involvement showed mixed responses with some cases being improved, some being worsened, and some being stable [6].

Although there have been some studies on cardiac manifestations in MPS patients and results of ERT for MPS patients including a few adult patients [[7], [8], [9], [10]], there has been no study conducted only for adult patients. We present here cardiac manifestations in four adult patients with Scheie syndrome who had been receiving ERT for over 10 years together with some literature reviews.

## 2 Methods

### 2.1 Subjects

The subjects were four adult patients (two males and two females) with Scheie syndrome who were receiving ERT in Kochi Medical School Hospital. The diagnosis of MPS I was based on the demonstration of a deficiency of α-IDUA activity in leukocytes. None of the patients underwent hematopoietic stem cell transplantation. This study was approved by the Ethical Review Board of Kochi Medical School, Kochi University.

### 2.2 Clinical evaluations

Evaluations included medical history, clinical examination, 12‑lead electrocardiography (ECG), M-mode echocardiography, 2-dimensional (2D) echocardiography, and Doppler echocardiography. Echocardiographies were evaluated before and 10 years after the initiation of ERT. Data were digitally stored and analyzed by cardiologists. The following parameters were measured by M-mode or 2D tracing: left ventricular end-diastolic dimension (LVEDD), left ventricular end-systolic dimension (LVESD), interventricular septum thickness (IVST), left ventricular posterior wall thickness (PWT), and left atrial dimension (LAD). For the assessment of valvular function, valvular peak gradients were calculated from continuous-wave Doppler using the simplified Bernoulli equation. The severity of valvular stenosis and regurgitation were estimated and graded as follows according to the American Society of Echocardiography guidelines: none, mild, moderate, and severe. Systolic pulmonary artery pressure was derived from tricuspid systolic gradient together with an estimate of right atrial pressure using inferior vena cava dimension.

## 3 Results

Clinical characteristics of the four patients are summarized in Table 1. Patient 2 and patient 4 are siblings. Patient 1 and patient 3 had an identical mutation: c.266G > A, p.R89Q homozygous mutation although these two patients were not relatives each other. The siblings of patient 2 and patient 4 did not want to have genetic analysis. All of the patients presented with joint contracture in their childhood. Although the ages at onset of MPS I were in their early childhood, their ages at diagnosis varied from 10 to 30 years. When they were diagnosed with Scheie syndrome, laronidase had not been approved in Japan. Therefore, they could not receive ERT just after they had been diagnosed with Scheie syndrome. After the approval of laronidase in Japan, the mean starting age of ERT was 36.7 years (26–46 years).

**Table 1:** Clinical features of four patients.

Patient	1	2	3	4
Sex	F	M	F	M
Age at onset (years)	4	3	6	3
Age at diagnosis (years)	30	14	30	10
Age at initiation of ERT (years)	46	29	46	26
Age at valve replacement (years)	44	28	54	−
Age at latest follow-up (years)	57	40	56	38
Follow-up period (months)	130	131	121	134
Gene mutation	p. R89Q (homo)	Not available	p. R89Q (homo)	Not available
Typical dysmorphic features	±	+	±	+
Joint contractures	+	+	+	+
Height (cm)	138 (−3.8SD)	167 (−0.9SD)	144 (−2.7SD)	156 (−2.9SD)
Visual disorder	+	+	+	+
Cognitive impairment	−	−	−	±

ERT; enzyme replacement therapy

The mean ERT period was 129 months (121 to 134 months) in this study. At the latest follow-up, the mean age of the four patients was 47.8 years (38–57 years). They received 0.58 mg/kg/week of intravenous laronidase.

In electrocardiographic findings, before starting ERT, no abnormalities were found on ECGs except for the ECG for patient 1, who had atrial fibrillation with a heart rate of 100 beats per minute. The other three patients had normal sinus rhythm, normal conduction time, and normal QRS complex. On the latest ECG, patient 1 showed atrial tachycardia and LV hypertrophy defined as SV1 + RV5 ≥ 3.5 mV (Sokolow-Lyon voltage criterion) and patient 2 showed bigeminy. Patient 1 and patient 3 had permanent pacemaker implantation for sick sinus syndrome with near syncope after their valve surgery.

**Fig. 1:**
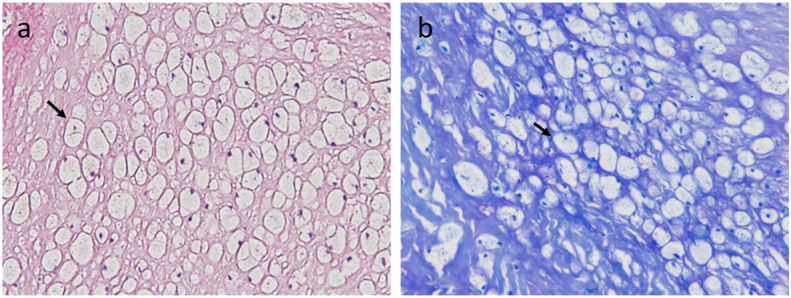
Histological images of resected valve. (a) Hematoxylin and eosin stain of aortic valves. Arrow indicates that a large number of foamy cells infiltrated in valve. (b) Toluidine blue stain of aortic valve. Arrow indicates glycosaminoglycan accumulation in the foamy cells.

Table 2 shows echocardiographic findings for the patients at the initiation of ERT and at the latest follow-up. At the start of ERT, no patients had LV systolic dysfunction and one patient (patient 1) had LV hypertrophy of posterior wall. The LV hypertrophy in patient 1 improved during ERT. No other significant changes in LV systolic function, LV diameter, and wall thickness were observed. Patient 1 and patient 2 had valve replacement before ERT: Patient 1, double valve replacement for severe aortic stenosis and severe mitral stenosis: and Patient 2, aortic valve replacement for severe aortic stenosis. While receiving ERT, tricuspid regurgitation progressed in patient 1 (none to mild). Pulmonary valve insufficiency did not progress. At eight years after the start of ERT, patient 3 had presyncope and valve studies demonstrated developing aortic and mitral stenoses, and the patient finally underwent double valve replacement and tricuspid annuloplasty. The resected valves in the surgery were thickened. The findings of the hematoxylin and eosin stain of these valves showed that a large number of foamy cells infiltrated in both valves. Toluidine blue and periodic acid shiff-stain positive granules accumulated in the cells (shown in Fig. 1). Little calcification was observed in aortic valve. These histological features were different from those of aging degeneration or rheumatic valvular disease. In patient 4, valvular disease did not progress. Regarding pulmonary hypertension, pulmonary hypertension that estimated by using echocardiography was detected in two patients (patients 1 and 3) at baseline. Echocardiography showed that patient 1 and patient 3 had mild pulmonary hypertension. At the preoperative evaluation of patient 3, the findings of right heart catherization showed that systolic pulmonary artery pressure was 29 mmHg and mean pulmonary artery pressure was 20 mmHg (<25 mmHg), although estimated systolic pulmonary pressure by using echocardiography performed at the same time was 43 mmHg. There was a gap between both values, and clinically remarkable pulmonary hypertension was not found. The other two patients did not show pulmonary hypertension.

**Table 2:** Echocardiographic findings before and after ten years ERT.

Patient	1		2		3		4	
	Before	Latest	Before	Latest	Before	Latest	Before	Latest
LVEDD (mm)	37	37	48	54	43	42	44	47
LVEDS (mm)	17	21	35	43	19	30	28	27
LVEF (%)	85	69	54	54	81	58	68	73
IVST (mm)	10	7	8	8	8	8	8	8
PWT (mm)	12	8	9	8	8	8	9	8
LAD (mm)	39	50	35	36	38	41	28	27
TrPG (mmHg)	32	42	17	17	34	20	18	20
Estimated RAP	3	3	3	3	15	8	3	3
Estimated PAP	35	45	20	20	49	28	21	23
AS	AVR	AVR	AVR	AVR	none	AVR	none	none
AR	AVR	AVR	AVR	AVR	mild	AVR	mild	none
MS	MVR	MVR	none	none	mild	MVR	none	none
MR	MVR	MVR	mild	mild	mild	MVR	none	mild
TR	none	mild	none	none	moderate	mild (TAP)	none	none

ERT; enzyme replacement therapy, LVEDD; left ventricular end-diastolic dimension, LVEDS; left ventricular end-systolic dimension, IVST; interventricular septum thickness, PWT; ventricular posterior wall thickness, LAD; left atrial dimension, TrPG; trans-tricuspid pressure gradient, RAP; right atrial pressure, PAP, pulmonary artery pressure, AS; Aortic stenosis, AR; aortic regurgitation, MS; mitral stenosis, MR; mitral regurgitation, TR; tricuspid regurgitation, AVR; aortic valve replacement, MVR; mitral valve replacement, TAP; tricuspid annuloplasty

During the follow-up period, no patient had decompensated heart failure. In the four patients, plasma brain natriuretic peptide values did not change significantly: from mean value of 42.4 pg/ml (7.8–77.5 pg/ml) at the start of ERT to mean value of 50.6 pg/ml (11.5–93.7 pg/ml) at the latest follow-up.

**Fig. 2:**
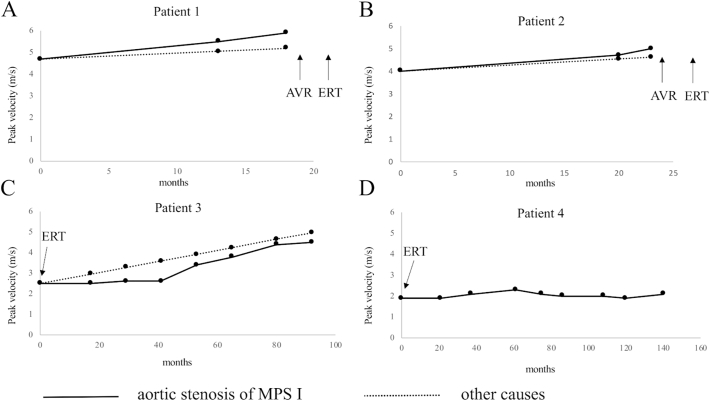
Time course of aortic valvular peak velocity in MPS I patients compared with that in patients with aortic stenosis of other causes. The solid line shows the transition of trans-aortic jet velocity in MPS I patients, and the dotted line shows the previously reported data demonstrating the rate of progression of aortic jet peak velocity in patients with aortic stenosis of other causes (0.32 ± 0.34 m/s per year) [11]. Before receiving ERT, patient 1 and patient 2.

Fig. 2 shows changes of trans-aortic valve peak velocity in the four patients compared with progressive rates of aortic stenosis due to other causes such as degeneration, bicuspid, and rheumatic fever. Otto et al. reported that aortic jet velocity increased by 0.32 ± 0.34 m/s per year in the patients with aortic stenosis including 71% of calcific, 28% of bicuspid, and 1% of rheumatic [11]. Patient 1 and patient 2 developed severe aortic stenosis before laronidase was approved in Japan (Fig. 2A and B). The rates of hemodynamic progression in patient 1 and patient 2 without ERT treatment were 0.75 m/s per year and 0.42 m/s per year, respectively. On the other hand, hemodynamic progression in patient 3 was 0.26 m/s per year (Fig. 2C). while receiving ERT, aortic jet velocity in patient 4 was stable (Fig. 2D).

## 4 Discussion

To the best of our knowledge, this is the first report on cardiac manifestations in adult patients with the attenuated form of MPS I (Scheie syndrome) who were treated with ERT for a period of 10 years.

The efficacy of ERT for cardiac disease varies from patient to patient and remains to be elucidated. Because there has been no natural cohort available for cardiac outcome in Scheie syndrome patients, we were not able to compare our data with the natural history in patients with the attenuated form of MPS I regarding the effectiveness of ERT. Braunlin et al. reported the efficacy of ERT for MPS I in children. They reported that mitral regurgitation increased in three of four patients and decreased in the other patient after long-term ERT. Aortic regurgitation increased in four of five patients and was stable in the other patient [12]. Laraway et al. also reported that valvular insufficiency occurred at a young age in MPS I patients. Patients whose ages ranged between 0.5 and 23.1 years (median age of 11.3 years) were included in their study. Valvular fuctions remained stable in 65% of the patients, deteriorated in 32% of the patients and improved in 3% of the patients during ERT. Deterioration of valvular functions occurred in fewer patients aged <10 years than in patients aged ≧ 10 years at the time of treatment initiation [6].

Three of our four patients had severe aortic stenosis and two of those three patients had moderate or severe mitral stenosis. Those patients underwent valve replacement. Patient 4 started ERT at the youngest age among the four patients and his valvular insufficiency did not progress. This is consistent with the results of other studies suggesting that ERT might be more effective in young patients [10], although these difference of clinical courses can be explained by the heterogeneous manifestations in patients with the attnuated form of MPS I.

Progression of aortic stenosis in patients with Scheie syndrome is unresolved. At the initiation of ERT, patient 1 and patient 2 had already developed significant valvular insufficiency. Patient 3 has developed to severe aortic valve stenosis and mild to moderate mitral valve stenosis during ERT. The rate of hemodynamic progression of aortic stenosis in patient 3 was slower than that in patient 1 and patient 2, who had developed severe valvular insufficiency before the start of ERT (Fig. 2). The rate of hemodynamic progression of aortic stenosis in patient 3 might also be slower than that in cases of aortic valve stenosis of other causes such as degeneration, bicuspid, and rheumatic fever [11]. In contrast, hemodynamic progression of aortic stenosis in patient 1 and patient 2, who had developed severe aortic stenosis before they started ERT, was faster than that in case of aortic stenosis of other causes.

In previous reports, microscopy of resected aortic valves from patients with Scheie syndrome showed numerous numbers of vaculated cells. Periodic acid-shiff stain-positive and alcian blue-positive cells were detected [13,14]. Similar findings were observed in our patient (Fig. 1). These findings indicated that accumulation of glycosaminoglycans leads to valve thickening and development of valvular insufficiency.

Our findings might suggest that aortic stenosis in MPS I patients progresses more rapidly than does aortic stenosis of other causes. Although there is little available evidence for assessing the effectiveness of treatment, ERT might decrease the rate of progression of valvular disease in patients with MPS I.

Two of our four patients developed sick sinus syndrome, resulting in permanent pacemaker implantation. Conduction abnormalities have been reported in subset of the Hunter Outcome survey of MPS II [15], in MPS III patients [16], and in MPS IV patients [17]. There has been no report about conduction abnormalities in MPS I patients. We consider that the development of sick sinus syndrome in patients 1 and 3 could have been a surgical issue rather than MPS itself.

Survival of patients with Scheie syndrome is clearly better than that of patients with Hurler syndrome [18]. Although Scheie syndrome is the mildest form of MPS I and patients survive to adolescence or adulthood, premature death in untreated MPS patients is most commonly a result of respiratory compromise and cardiac disease [19]. In this study, although two patients developed sick sinus syndrome after valvular surgery and underwent permanent pacemaker implantation, all of the three patients who had prosthetic valve replacement were followed with an uneventful course. Thus, valvular replacement therapy might prevent premature death in patients with Scheie syndrome. However, this study had a small sample size and was a retrospective study. There remains unanswered clinical questions and further works should be done in this field.

## 5 Conclusions

Valvular disease in patients with the attenuated form of MPS I occurs at a young age. In a limited number of the four adult patients who were treated with ERT for a period of 10 years, their clinical courses were relatively stable and ERT might contribute the stability of cardiac condition.

## Funding

This research did not receive any specific grant from funding agencies in the public, commercial, or not-for-profit sectors.

## Declaration of Competing Interest

None of the authors have conflict of interest to disclose in connection with our manuscript.

(A and B) had already undergone valve replacement. Their rates of hemodynamic progression were 0.75 m/s per year (patient 1) and 0.42 m/s per year (patient 2). Patient 3 (C) developed aortic valve stenosis during ERT. The rate of progression was showed 0.26 m/s per year (C). *trans*-Aortic valve peak velocity in patient 4 was stable during ERT (D). MPS: mucopolysaccharidosis, AVR: aortic valve replacement, ERT: enzyme replacement therapy.
